# Multi-level Factors Influencing Decisions About Stopping Surveillance Colonoscopy in Older Adults: a Qualitative Study

**DOI:** 10.1007/s11606-023-08225-0

**Published:** 2023-05-24

**Authors:** Karen E. Schifferdecker, Nithya Puttige Ramesh, Louise C. Walter, Audrey H. Calderwood

**Affiliations:** 1 The Dartmouth Institute for Health Policy and Clinical Practice at Geisel School of Medicine, Lebanon, NH USA; 2 Center for Program Design and Evaluation at Dartmouth, NH Lebanon, USA; 3 School of Public Health, Brown University, Providence, RI USA; 4 Division of Geriatrics, University of California San Francisco and San Francisco VA Health Care System, San Francisco, CA USA; 5 Section of Gastroenterology, Dartmouth-Hitchcock Medical Cancer, Lebanon, NH USA

**Keywords:** colonoscopy, surveillance, older adults, qualitative

## Abstract

**Background:**

Little is known about patient or provider experience and perceptions of stopping surveillance among older adults with a history of colon polyps. While guidelines recommend ceasing routine colorectal cancer screening in adults  > 75 years and those with limited life expectancy, guidance for ceasing surveillance colonoscopy in those with prior colon polyps suggests individualizing recommendations.

**Objective:**

Identify processes, experiences, and gaps around individualizing decisions to stop or continue surveillance colonoscopy for older adults and areas for improvement.

**Design:**

Phenomenological qualitative study design using recorded semi-structured interviews from May 2020 through March 2021.

**Participants:**

15 patients aged  ≥ 65 in polyp surveillance, 12 primary care providers (PCPs), and 13 gastroenterologists (GIs).

**Approach:**

Data were analyzed using a mixed deductive (directed content analysis) and inductive (grounded theory) approach to identify themes related to stopping or continuing surveillance colonoscopies.

**Key Results:**

Analysis resulted in 24 themes and were clustered into three main categories: health and clinical considerations; communication and roles; and system-level processes or structures. Overall, the study found support for discussions around age 75–80 on stopping surveillance colonoscopy with considerations for health and life expectancy and that PCPs should take a primary role. However, systems and processes for scheduling surveillance colonoscopies largely bypass PCPs which reduces opportunities to both individualize recommendations and facilitate patients’ decision-making.

**Conclusions:**

This study identified gaps in processes to implement current guidelines for individualizing surveillance colonoscopy as adults grow older, including opportunities to discuss stopping. Increasing the role of PCPs in polyp surveillance as patients grow older provides more opportunities for individualized recommendations, so patients can consider their own preferences, ask questions, and make a more informed choice for themselves. Changing existing systems and processes and creating supportive tools for shared decision-making specific to older adults with polyps would improve how surveillance colonoscopy is individualized in this population.

## INTRODUCTION

Current guidelines recommend that persons diagnosed with pre-neoplastic adenomas and serrated polyps during colonoscopy have ongoing surveillance colonoscopies at intervals of 3–7 years since they are at higher risk of developing colorectal cancer.^1^ While surveillance after polypectomy is widely practiced in the USA, there are limited data on whether surveillance colonoscopy reduces CRC incidence or mortality,^2,3^ with even less data in older adults.^4^ More older adults, including those with limited life expectancy,^5,6^ are being enrolled in colonoscopy surveillance protocols (estimated 6 million annually^7^) because of increased screening and improved polyp detection.^8,9^ However, potential harms of colonoscopy, such as risk of bleeding, perforation, and cardiopulmonary complications, increase with age^10–12^ supporting the importance of weighing potential benefits and harms when making surveillance colonoscopy recommendations to older adults.

While guidelines recommend against routine screening colonoscopies in adults  > 75 years and those with limited life expectancy,^13^ there is a paucity of guidance on when surveillance colonoscopy for history of polyps should stop,^1,14^ and a lack of “detailed, age-specific data on risks and benefits” for older adults to develop more specific recommendations.^15^ As such, the US Multi-Society Task Force recommends that “the decision to continue surveillance be individualized based on benefits, risks, and co-morbidities,”^14^ but how this is implemented in practice is unknown.

Prior studies have found that patients want to be involved in decisions regarding surveillance colonoscopy^16^ and that primary care providers (PCPs) use a range of approaches to decide about continuing or stopping surveillance colonoscopies.^17^ More in-depth perspectives from patients, PCPs, and specialists regarding decision-making, processes of care, and communication around stopping surveillance colonoscopy would assist in understanding current implementation of guidelines and areas for improvement. The aim of this qualitative study was to comprehensively assess patient, PCP, and gastroenterologist (GI) perceptions around stopping polyp surveillance in older adults, including considerations, communication, roles, and care processes around surveillance.

## METHODS

### Study Design and Criteria

We chose a phenomenological qualitative study design, which emphasizes close examination of individual experiences to capture the “meaning and common features” of those experiences.^18^ Given our focus on three angles of the experience of surveillance colonoscopy, we used semi-structured interviews. The study was approved by the Institutional Review Board at Dartmouth-Hitchcock Medical Center as minimal risk with waiver of signed informed consent and was conducted between May 2020 and March 2021. We follow the consolidated criteria for reporting qualitative studies (COREQ).^19^

### Participants and Inclusion Criteria

We established the following criteria for study participants to ensure lived experience of surveillance colonoscopy:

- GIs—participated in the New Hampshire Colonoscopy Registry (NHCR), a state-wide population-based colonoscopy registry^20,21^ with screening and surveillance colonoscopy data in the NHCR within the past 5 years and performed at least 100 colonoscopies annually.
- PCPs—Family Medicine or Internal Medicine–trained MDs, DOs, nurse practitioners, or physician assistants who care for older adults in New Hampshire.
- Patients—English-speaking, aged  ≥ 65, undergoing surveillance colonoscopy, no prior colorectal cancer, and cognitively able to participate in an interview.

### Sampling and recruitment

We use purposive sampling and varied recruitment methods which included the following steps:

- GIs—A letter regarding the study was sent to NHCR sites, including academic centers, community hospitals, and private groups requesting permission to contact their providers. Once permission was obtained, an email was sent inviting providers to participate.
- PCPs—PCPs were recruited from an academic center, practice-based research network affiliates, and private groups after a presentation at their staff meeting by a study team member followed up with a general email invitation and/or direct email invitation.
- Patients—Eligible patients were referred by their PCPs and GIs and also directly recruited through advertisements in clinic waiting rooms. Interested participants completed a brief recruitment survey focused on demographics and eligibility criteria and were then contacted for an interview. We aimed to include patients of varying genders, socioeconomic and educational backgrounds, and regions (urban/rural).

### Research Team

Our research team included two physicians (one PCP, one GI), a medical anthropologist and mixed-methods researcher, and a trained dentist with mixed-methods and public health training. While the physicians were involved in designing the research, including interview guide development and data analysis review, interviews were conducted by the non-physicians to encourage more explanation of care processes by PCP and GI participants and of experiences of care by patient participants.

### Interviews

We developed three interview guides to customize questions relevant for each of the participant groups all of which covered similar domains related to our research question (Table 1). Questions were based on prior literature on surveillance^22–25^ and the aims of the study. The two non-physician authors (K.E.S., N.P.R.) conducted the interviews over videoconferencing. Sessions lasted 30–60 min and were recorded and transcribed. All participants were offered a $50 gift card in appreciation of their time.

**Table 1:** Domains Covered in the Interview Guides for Gastroenterologists, Primary Care Providers, and Patients Regarding Stopping Surveillance Colonoscopy

Gastroenterologists	Primary care providers	Patients
Process related to initiating the colonoscopy request and continuing surveillance colonoscopy		
Understanding and views about guidelines for surveillance colonoscopy		Understanding of polyps and risk of colon cancer
Reliance/trust with PCPs making surveillance recommendations, including stopping	Reliance/trust with GIs making surveillance recommendations, including stopping	Frequency, types of recommendations or communications they have received and from whom
Considerations and process related to stopping surveillance colonoscopies		Response to recommendations to stop surveillance

### Data Analysis and Interpretation

Transcripts were uploaded to Dedoose.^26^ Two authors (K.E.S., N.P.R.) developed the codebook using a mixed deductive (directed content analysis) and inductive (grounded theory) approach.^27–29^ Specifically, we pre-determined deductive codes based on the main research questions and our phenomenological approach related to processes, experiences, and considerations for continuing or stopping surveillance colonoscopy (e.g., age, life expectancy, provider communication) and developed additional inductive codes based on iterative review of the data. The initial inductive codes were developed through joint coding of one interview for each participant type and then reviewed by one additional author (A.H.C.) and revised to incorporate feedback. N.P.R. then coded an additional transcript for each participant type, K.E.S. reviewed changes to the codes, and then N.P.R. coded all additional transcripts with periodic coding checks by K.E.S. Coding changes (e.g., revised definitions, merges) were systematically tracked, disagreements were discussed and resolved through consensus, and saturation was assessed within each participant group by noting when no new codes were added. K.E.S. and N.P.R. then grouped the codes into themes and discussed these with A.H.C. and L.C.W. to reach congruency. The final code book consisted of 179 codes. We used descriptive statistics to summarize the characteristics of the study participants.

## RESULTS

We interviewed 13 GIs, 12 PCPs, and 15 patients. Table 2 describes the characteristics of our sample. Among the GIs, four (31%) were female and their mean number of years in practice was 17. Among the PCPs, eight (66%) were female and their mean number of years in practice was 19. Among the patients, eight (53%) were female, and 5 (33%) were  ≥ 75. Most (73%) had attended college.

**Table 2:** Characteristics of the Gastroenterologist, Primary Care Provider, and Patient Participants

	GIs	PCPs	Patients
Number of participants	13	12	15
Female, *n* (%)	4 (31)	8 (66)	8 (53)
Provider characteristics			
Years in clinical field, mean (range)	17 (6–29)	19 (1–38)	–
Years at current practice, mean (range)	15 (4–25)	6.5 (1–16)	–
Affiliated with same medical system, %	62	80	–
No. of surveillance colonoscopies performed per week on average	5–45	–	–
Additional patient characteristics			
Age, years, *n* (%)	–	–	
65–74			10 (67)
≥ 75			5 (33)
Educational attainment	–	–	
High school or less			4 (27)
College or higher			11 (73)
Most recent surveillance interval	–	–	
3 years			7 (46)
5 years			6 (40)
10 years			2 (14)

**Figure 1:**
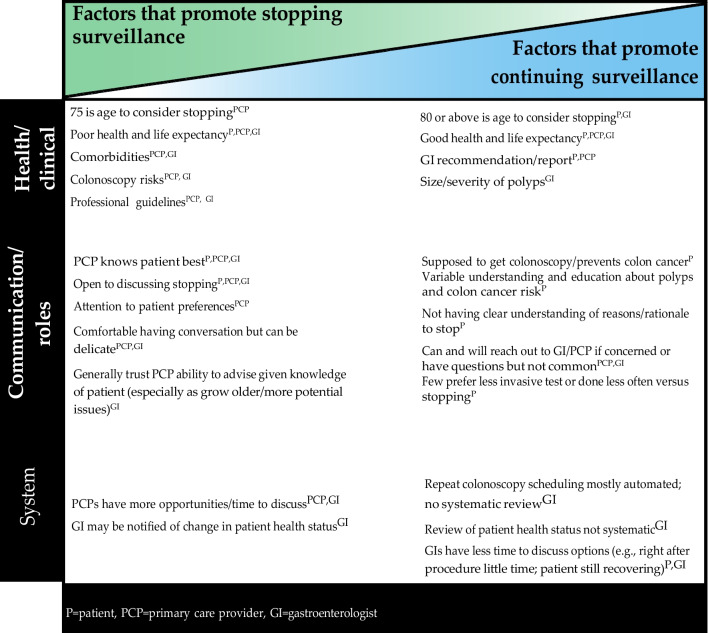
Themes that promoted stopping or continuing surveillance.

We achieved saturation by interview 11 with patients, 10 with PCPs and 9 with GIs. While we could have ceased interviewing additional participants after this point, ongoing scheduling in conjunction with coding resulted in the additional interviews. Through cross-comparisons during analysis, we identified 24 themes that promoted stopping or continuing surveillance which clustered into three main categories (see Fig. 1): (1) health and clinical considerations; (2) communication and roles; and (3) system-level processes or structures. As indicated, some themes were specific to one group (e.g., patients) and others were related to two or all three participant groups. Below we provide an overall summary of themes in each category and example quotes.

### Themes in Health and Clinical Considerations

Nine themes emerged in health and clinical considerations. Three of the five themes that promoted stopping surveillance colonoscopies centered on poor health or life expectancy. PCPs and GIs then often tied these to the related themes of increased colonoscopy risks particularly for patients with comorbidities. On the flip side, all three groups highlighted good health and life expectancy as a reason to continue surveillance.

*PCP: Once in a while, you might have somebody who’s over 75, who’s super healthy, and if you had to predict... mortality, you would say that it would be reasonable for them to continue surveillance.*

All groups also viewed GI recommendations, mostly in the form of the GI colonoscopy report, as a key influence to continue, with GIs mentioning size and severity of polyps as an influence on their recommendations.

Patient age was a theme across all three groups and appeared to both promote and hinder considerations for stopping surveillance. PCPs often cited 75 as the age to start conversations related to whether to continue while patients and GIs more often mentioned 80 or above.

*PCP: So that usually happens around the age 75, and it’s a little bit their [patients], how they think about, how they view the possibility of having cancer is one factor. Their thinking about the risk and inconvenience of colonoscopy and the potential benefit of finding cancer against their projected life expectancy. And also explain to them that from polyps to cancer, it takes a long time… So if they’re 75 or older, they have to think of how they think about their longevity and whether they want to undergo a procedure that could benefit them, but that also has to carry some small risk.*

*Patient: I’m 76, so I figure in the next 10 years, that’s okay, I can get them. I think... When I’m 85 or 90, I don’t know that I’ll get them anymore. I mean, I’ve thought about this. It feels as though at some point, that if it’s cancer it’ll be slow enough that I’d probably won’t want an operation.*

*GI: We don’t have any strict age criteria per se. 85 is kind of our cutoff.*

While PCPs and GIs cited professional guidelines, these may have variable influence for considering stopping surveillance.

*GI: We practice based on guidelines, but everything is individualized. The guideline doesn’t tell you, “Well, this patient has these multiple comorbidities. They’re going to say yes or no.” You have to practice within the guideline, but it’s a guideline. It tells you what you should do, but you can modify it based on the presenting situation and patients.*

### Themes in Communication and Roles

Ten themes emerged in communication and roles. Considering those that promoted stopping surveillance, all three groups expressed a willingness to have conversations with each other about stopping and felt that PCPs were in a good position to advise patients about stopping as they “know” patients best (e.g., better personal relationship, knowing overall health).

*Patient: My PCP knows me, knows my history, and knows me better. I’ve been with this doctor now 10 years since we moved here. I would want to get her perspective; it’s not that it would override the gastroenterologist’s. It’s just I would like to get her perspective as well, so that I can make a good decision.*

*PCP: So, I’m their primary care doctor, I’m not just a gastroenterologist. So, for me, I think knowing your patient, having a relationship, knowing what their goals are, knowing what their risk factors are, and knowing what they’re going through in life is very, very important.*

PCPs and GIs generally suggested that PCPs’ role in advising patients about surveillance increased as patients grow older or develop new health conditions. Some GIs described this as deferring to the PCP regarding the patient stopping or continuing surveillance colonoscopies.

*GI-A: They see the patient a couple of times a year and they know how they’re doing, how…they’re functioning…ability to cope with the day in and day out stresses of life and, and medical care. So, yeah, I’m pretty deferential to the patient and the primary care physician.*

*GI-B: If I see that they’re going to be reaching that age with their next procedure due date, then I actually put it into the letter and talk to the patient about it that you’re going to be advanced age, so you need to have a discussion with your primary care doctor at that point about your overall health status and make that decision.*

Although PCPs and GIs appeared to respect each other’s roles around surveillance and felt able to reach out with questions or concerns, both groups described this as a rare occurrence likely resulting in continuing surveillance more often than not.

In terms of communication with patients, both PCPs and GIs felt comfortable having conversations about stopping surveillance, with PCPs bringing up patient preferences more often. At the same time, both groups acknowledged it could be a “delicate” discussion particularly in relation to age and life expectancy.

*GI: Really, it’s a difficult area to talk about because it has a lot to do with mortality. What you’re really saying to somebody is, well, you’re going to die of something else, so let’s not bother with colonoscopy. But you can’t really say that because that doesn’t go over well, but that’s kind of what you’re saying. I think it sort of depends. Some patients take it as “great, I don’t need another colonoscopy.” Others take it as, well, “you’re not taking care of me because I’m too old,” so it’s a pretty delicate topic.*

In the themes that appear to promote continuation of surveillance, four of the five were patient-specific, including patients feeling they are “supposed” to continue to prevent colon cancer, patients having variable understanding and education about polyps and colon cancer risk, and questioning reasons to stop, with some preferring switching to a less invasive test or delaying a colonoscopy versus stopping altogether.

*Patient: I guess if there were valid reasons, I could accept it, but I’d want to know what those reasons are.*

#### Themes Related to Systems

We identified five main themes related to system-level processes with two that appeared to promote stopping surveillance and three that did not. PCPs and GIs both noted that PCPs have more opportunities to discuss surveillance given more frequent visits and time during visits. Conversely, patients and GIs noted that GIs do not have opportune times to really discuss options given that interactions usually take place right after colonoscopies for a short period and when patients might still feel effects of colonoscopy sedation.

GIs also noted other processes that promote continued surveillance such as automated scheduling of subsequent surveillance colonoscopies. While a few GIs described making a note in the scheduling system to check on the patient’s status before scheduling if they had concerns with the patient’s health or that they were sometimes notified of a change in patient health status by another healthcare team member, this was not systematic or regularly done.

*GI: I just say follow up in five years. Somehow that magically happens, and I don’t know how. Somebody puts that into the scheduling system.*

## DISCUSSION

In this study regarding decision-making around surveillance colonoscopy in older adults, we found general openness to the concept of stopping surveillance. All three participant groups agreed on important influencing factors, specifically age (75–80) and comorbidities and, additionally for GIs, polyp characteristics. While GI recommendations were acknowledged as key for deciding what to do, there was general agreement that PCPs are in the best position to advise on stopping surveillance, particularly as patients grow older. However, we found system processes that promote continued surveillance without routine review of changes in patient health or discussion of risks, benefits, and patient preferences, which go against current guidelines to individualize surveillance colonoscopy in older adults.

This study adds valuable information to the existing yet limited literature on this topic and differs in finding support for PCPs having a more primary role. Our findings are similar to a study of PCP decision-making around surveillance colonoscopies in older adults that found most PCPs based decisions on age, comorbidities, or life expectancy,^17^ all of which may change the balance of risk and benefits for continued surveillance. Some PCPs took on discussions with the patient directly, similar to our study, and some PCPs deferred more to the GI due to discomfort in making decisions. In a different study on low-risk adenoma surveillance decision-making in general (not specifically older adults), PCPs had varying perceptions of their role.^30^ Overall, they felt more involved in screening decisions than surveillance and that surveillance decisions should be shared between PCPs and GIs or between PCPs and patients while GIs believed surveillance was their purview. Our study, in contrast, suggests both PCPs and GIs were open to a more expanded role for PCPs in relation to stopping surveillance. In addition, we found that while PCPs and GIs respect each other’s expertise and role in decision-making around surveillance, there is a lack of inter-specialty communication. This is similar to findings of a 2017 survey in which 70% of PCPs mentioned never being contacted by GIs regarding colonoscopy in older adults.^31^

Considering views of patients, we found similar results to a study focused on surveillance colonoscopy with older adults around patients trusting and relying on PCPs and wanting to base decisions to stop surveillance on overall health versus age alone.^16^ However, the study participants also acknowledged more turnover in PCPs and seeing multiple different providers in the same practice as barriers to building trust. Even so, participants in our study suggested PCPs as a whole still have more potential opportunities (e.g., annual or sick visits) and time to discuss risks and benefits with patients, and might consider overall health, life situation, and patient preferences more.

Our study highlights several potential ways to improve care of older adults with a history of polyps facing decisions about surveillance colonoscopy. As patients age and/or develop comorbidities, PCPs should be prepared to be the main drivers of discussions on individualizing surveillance decisions. Post-colonoscopy recommendations and reports could also be improved by suggesting that PCPs and patients discuss if surveillance should continue or not. For example, instead of saying “recommend surveillance colonoscopy in 5 years,” an alternative could be “recommend PCP and patient discuss surveillance colonoscopy in 5 years,” which promotes up-to-date shared decision-making when the next colonoscopy is suggested.^15^ This is particularly important given PCPs often rely on EHR alerts and GI recommendations.^30^ There is also an opportunity for more involvement on the endoscopy system’s side to move away from the current default of automatic rescheduling regardless of age. For example, parameters could be put in place based on patient age (e.g.,  ≥ 70 years old) to trigger a more formal review of the patient’s health status and/or recommend the patient and PCP discuss prior to scheduling. In addition, systems could be put into place to facilitate inter-specialty communication where PCPs can get expert advice from GIs as needed (e.g., e-consults).

Other frameworks for advising older adults on cancer screening in general^32^ and surveillance in particular^15^ include specific recommendations for developing and using tools such as estimates of life expectancy, cancer death risks, and screening outcomes for providers and decision aids for patients to support individualized estimates and counseling. While a few of our PCP participants mentioned use of specific data or tools, most spoke more generally about life expectancy and comorbidities. Nevertheless, the development of more specific data, tools, and decision aids specific to older adults with polyps should benefit discussions and decisions related to stopping or continuing surveillance colonoscopy.

The main strength of our study is the inclusion and comparison of perspectives across patients, PCPs, and GIs. The finding that all three groups agreed that PCPs are in the best position to provide guidance supports developing better systems, including allocated time, specific data, and tools, to facilitate this. We acknowledge certain limitations. Our study had heavy representation from one academic center and other providers who participated in the NHCR, where they may be more engaged in quality improvement and knowledgeable of evidence. Given that we were based primarily in one state, we cannot comment on potential regional variation. Patient views may not be representative of older adults elsewhere or those with less educational attainment or without regular medical care. However, qualitative studies are designed to gain rich, in-depth information in an area with little prior knowledge and our study procedures and findings can be replicated and tested in further studies.

## CONCLUSIONS

We found support for discussions about stopping surveillance colonoscopy around age 75–80 with considerations for health and life expectancy and for PCPs taking a primary role in these discussions. However, existing systems would need to be changed to better support PCPs’ role in facilitating patients’ shared decision-making. Such changes could empower PCPs to take on more of a lead role and provide an opportunity for patients to consider their own preferences, to ask questions, and to make a more informed choice for themselves.

## Data Availability

Original de-identified data and codebook are available from the corresponding author.
